# Factors Associated With Survival Disparities Between Non-Hispanic Black and White Patients With Uterine Cancer

**DOI:** 10.1001/jamanetworkopen.2023.8437

**Published:** 2023-04-17

**Authors:** Calen W. Kucera, Chunqiao Tian, Christopher M. Tarney, Cassandra Presti, Suzanne Jokajtys, Stuart S. Winkler, Yovanni Casablanca, Nicholas W. Bateman, Paulette Mhawech-Fauceglia, Lari Wenzel, Chad A. Hamilton, John K. Chan, Nathaniel L. Jones, Rodney P. Rocconi, Timothy D. O’Connor, John H. Farley, Craig D. Shriver, Thomas P. Conrads, Neil T. Phippen, G. Larry Maxwell, Kathleen M. Darcy

**Affiliations:** 1 Gynecologic Cancer Center of Excellence, Department of Gynecologic Surgery and Obstetrics, Uniformed Services University of the Health Sciences, Walter Reed National Military Medical Center, Bethesda, Maryland; 2 John P Murtha Cancer Center Research Program, Department of Surgery, Uniformed Services University of the Health Sciences, Walter Reed National Military Medical Center, Bethesda, Maryland; 3 The Henry M Jackson Foundation for the Advancement of Military Medicine, Inc, Bethesda, Maryland; 4 Department of Obstetrics and Gynecology, Inova Fairfax Hospital, Falls Church, Virginia; 5 Aurora Diagnostics, LMC Pathology Services, Las Vegas, Nevada; 6 Department of Medicine, School of Medicine, University of California, Irvine; 7 Department of Public Health, School of Medicine, University of California, Irvine; 8 Gynecologic Oncology Section, Women’s Services and The Ochsner Cancer Institute, Ochsner Health, New Orleans, Louisiana; 9 Gynecologic Oncology Division, Palo Alto Medical Foundation/California Pacific Medical Center/Sutter Health, San Francisco; 10 Division of Gynecologic Oncology, the Mitchell Cancer Institute, University of South Alabama, Mobile; 11 Division of Gynecologic Oncology, the University of Alabama at Birmingham, Infirmary Cancer Care, Infirmary Health, Mobile; 12 Institute for Genome Sciences, Department of Medicine, Program in Personalized and Genomic Medicine, University of Maryland School of Medicine, Baltimore; 13 Program in Health Equity and Population Health, University of Maryland School of Medicine, Baltimore; 14 The University of Maryland Marlene and Stewart Greenebaum Comprehensive Cancer Center, Baltimore; 15 Division of Gynecologic Oncology, Dignity Health Cancer Institute, Dignity Health St Joseph’s Hospital and Medical Center, Phoenix, Arizona; 16 Department of Surgery, Uniformed Services University of the Health Sciences, Walter Reed National Military Medical Center, Bethesda, Maryland; 17 Women’s Health Integrated Research Center, Inova Women’s Service Line, Inova Health System, Falls Church, Virginia

## Abstract

**Importance:**

Disparities in survival exist between non-Hispanic Black (hereafter, Black) and non-Hispanic White (hereafter, White) patients with uterine cancer.

**Objective:**

To investigate factors associated with racial disparities in survival between Black and White patients with uterine cancer.

**Design, Setting, and Patients:**

This cohort study used data from the National Cancer Database on 274 838 Black and White patients who received a diagnosis of uterine cancer from January 1, 2004, to December 31, 2017, with follow-up through December 2020. Statistical analysis was performed in July 2022.

**Main Outcomes and Measures:**

Overall survival by self-reported race and evaluation of explanatory study factors associated with hazard ratio (HR) reduction for Black vs White patients. A propensity scoring approach was applied sequentially to balance racial differences in demographic characteristics, comorbidity score, neighborhood income, insurance status, histologic subtype, disease stage, and treatment.

**Results:**

The study included 32 230 Black female patients (mean [SD] age at diagnosis, 63.8 [10.0] years) and 242 608 White female patients (mean [SD] age at diagnosis, 63.5 [10.5] years) and had a median follow-up of 74.0 months (range, 43.5-113.8 months). Black patients were more likely than White patients to have low income (44.1% vs 14.0%), be uninsured (5.7% vs 2.6%), present with nonendometrioid histologic characteristics (46.1% vs 21.6%), have an advanced disease stage (34.1% vs 19.8%), receive first-line chemotherapy (33.8% vs 18.2%), and have worse 5-year survival (58.6% vs 78.5%). Among patients who received a diagnosis at younger than 65 years of age, the HR for death for Black vs White patients was 2.43 (95% CI, 2.34-2.52) in a baseline demographic-adjusted model and 1.29 (95% CI, 1.23-1.35) after balancing other factors. Comorbidity score, neighborhood income, insurance status, histologic subtype, disease stage, treatment, and unexplained factors accounted for 0.8%, 7.2%, 11.5%, 53.1%, 5.8%, 1.2%, and 20.4%, respectively, of the excess relative risk (ERR) among the younger Black vs White patients. Among patients 65 years or older, the HR for death for Black vs White patients was 1.87 (95% CI, 1.81-1.93) in the baseline model and 1.14 (95% CI, 1.09-1.19) after balancing other factors. Comorbidity score, neighborhood income, insurance status, histologic subtype, disease stage, treatment, and unexplained factors accounted for 3.0%, 7.5%, 0.0%, 56.2%, 10.6%, 6.9%, and 15.8%, respectively, of the ERR among Black vs White patients aged 65 years or older.

**Conclusions and Relevance:**

This study suggests that histologic subtype was the dominant factor associated with racial survival disparity among patients with uterine cancer, while insurance status represented the main modifiable factor for women younger than 65 years. Additional studies of interactions between biology and social determinants of health are merited.

## Key Points

**Question:**

What factors are most associated with racial disparities in survival between non-Hispanic Black and non-Hispanic White patients with uterine cancer?

**Findings:**

In this cohort study of 274 838 patients from the National Cancer Database, histologic subtype was associated with 54% of the racial disparities in survival. Insurance status was the major modifiable factor among those who received a diagnosis of uterine cancer at an age younger than 65 years.

**Meaning:**

This study suggests that histologic subtype was the dominant factor associated with racial disparities in survival among Black and White patients with uterine cancer.

## Teaser

This cohort study uses data from the National Cancer Database to investigate factors associated with racial disparities in survival between Black and White patients with uterine cancer.

## Introduction

The incidence and mortality of uterine cancer are increasing, with 66 570 new cases, including endometrial cancers and sarcomas, and 12 940 deaths estimated in the United States.^1,2,3,4^ Disparities between Black and White patients with uterine cancer have persisted over decades, with higher likelihoods of aggressive disease, recurrence, and death among Black patients.^5,6,7,8,9,10,11,12,13,14,15,16^ The factors explaining these trends and disparities are complex and multifactorial.^7,8,9,10,11,15,17,18,19,20,21,22^ Structural determinants of health integrate social constructs such as race and ethnicity with biological associations of inherited ancestry, lifestyle, exposures, and other factors to influence trends and disparities in disease susceptibility, severity, and outcomes.^17,18,20,21,22^ Even among patients with uterine cancer who have equal access to care^23^ and receive similar guideline-adherent treatment, racial differences in cancer diagnosis and survival persist.^15,24,25^ Differences in histologic subtype, disease stage, tumor grade, treatment, supportive services, and molecular features have been implicated in health disparities among patients with uterine cancer.^6,7,8,9,10,11,12,13,14,15,16,24,26,27,28,29,30,31,32,33,34,35,36,37,38,39,40,41,42,43,44,45,46,47,48,49,50^

Jemal et al^51^ illustrated the application of sequential propensity score matching with reductions in excess relative risk (ERR) to demonstrate that insurance status was the largest factor associated with racial disparities in survival among nonelderly patients with breast cancer. We questioned whether a modifiable factor, such as insurance status, or other factors are associated with survival disparities in uterine cancer. Our observational study used clinical data from Commission on Cancer (CoC)–accredited hospitals to investigate survival by self-reported race and ethnicity and explanatory factors associated with disparities between non-Hispanic Black (hereafter, Black) and non-Hispanic White (hereafter, White) patients with a diagnosis of stage I to IV uterine cancer.

## Methods

### Study Population

Data for this cohort study were acquired through a restricted access application process from the National Cancer Database (NCDB).^52,53^ Analyses used the 2021 NCDB Participant User File for patients who received a diagnosis between January 1, 2004, and December 31, 2017, with follow-up data through December 2020. Eligible Black or White female patients were required to have a diagnosis of stage I to IV uterine cancer. Black and White patients were defined based on the 2 self-reported variables coded by the NCDB, race and Hispanic or Spanish ethnicity. eFigure 1 in Supplement 1 shows the schema for the selection of Black and White patients with uterine cancer for this investigation of survival disparities. The clinical variables and criteria for both inclusion and exclusion are described in the table notes to Table 1. This study followed the Strengthening the Reporting of Observational Studies in Epidemiology (STROBE) reporting guideline and received an exemption of consent determination under Protocol 14-1679 by the Western Copernicus Group institutional review board because the data were deidentified.

**Table 1.** Clinical Characteristics of Non-Hispanic White and Non-Hispanic Black Patients With Uterine Cancer and Associations With Survival

Clinical characteristic	Difference by race and ethnicity^a^			Association with survival			
	Cases, No. (%)		*P* value	Unadjusted HR (95% CI)^b^	*P* value	Adjusted HR (95% CI)^c^	*P* value
	Non-Hispanic White	Non-Hispanic Black					
Race and ethnicity^d^							
White	242 608 (100)	NA	NA	1 [Reference]	<.001	1 [Reference]	<.001
Black	NA	32 230 (100)		2.11 (2.07-2.14)		1.22 (1.20-1.25)	
Demographic characteristics^e^							
Age at diagnosis, mean (SD), y	63.5 (10.5)	63.8 (10.0)	<.001	1.35 (1.34-1.35)	<.001	1.25 (1.24-1.25)	<.001
Census division							
New England	18 205 (7.5)	819 (2.5)	<.001	1 [Reference]	NA	1 [Reference]	NA
Middle Atlantic	40 606 (16.7)	5889 (18.3)		1.17 (1.13-1.21)	<.001	0.98 (0.95-1.02)	.32
East North Central	46 614 (19.2)	11 455 (35.5)		1.31 (1.26-1.35)	<.001	1.12 (1.08-1.16)	<.001
West North Central	47 818 (19.7)	4856 (15.1)		1.19 (1.15-1.23)	<.001	1.11 (1.07-1.15)	<.001
South Atlantic	15 517 (6.4)	3327 (10.3)		1.34 (1.29-1.39)	<.001	1.21 (1.17-1.26)	<.001
East South Central	21 951 (9.1)	1071 (3.3)		1.14 (1.10-1.19)	<.001	1.09 (1.05-1.13)	<.001
West South Central	14 093 (5.8)	2938 (9.1)		1.18 (1.13-1.23)	<.001	0.98 (0.94-1.02)	.31
Mountain	9709 (4.0)	232 (0.7)		1.07 (1.02-1.13)	.005	1.11 (1.06-1.17)	<.001
Pacific	28 095 (11.6)	1643 (5.1)		1.05 (1.02-1.09)	.006	1.04 (1.00-1.08)	.046
Year of diagnosis							
2004	11 512 (4.8)	1224 (3.8)	<.001	1 [Reference]	NA	1 [Reference]	NA
2005	12 259 (5.1)	1240 (3.9)		1.02 (0.98-1.06)	.31	1.06 (1.00-1.08)	.004
2006	13 089 (5.4)	1459 (4.5)		0.93 (0.90-0.97)	<.001	0.98 (0.95-1.03)	.27
2007	13 808 (5.7)	1579 (4.9)		0.94 (0.91-0.98)	.002	0.99 (0.95-1.03)	.51
2008	14 917 (6.2)	1739 (5.4)		0.95 (0.91-0.99)	.009	0.98 (0.95-1.03)	.27
2009	15 547 (6.4)	1869 (5.8)		0.93 (0.90-0.97)	<.001	0.98 (0.94-1.01)	.23
2010	17 774 (7.3)	2267 (7.0)		0.97 (0.94-1.01)	.18	0.99 (0.96-1.04)	.68
2011	18 603 (7.7)	2514 (7.8)		1.00 (0.96-1.04)	.95	1.01 (0.95-1.03)	.49
2012	19 373 (8.0)	2639 (8.2)		0.99 (0.95-1.03)	.49	0.94 (0.89-0.97)	.002
2013	20 359 (8.4)	2805 (8.7)		0.98 (0.94-1.02)	.24	0.92 (0.90-0.98)	<.001
2014	20 883 (8.6)	3120 (9.7)		0.96 (0.93-1.00)	.07	0.92 (0.91-1.00)	<.001
2015	21 216 (8.7)	3171 (9.8)		0.93 (0.89-0.97)	<.001	0.88 (0.85-0.92)	<.001
2016	21 566 (8.9)	3232 (10.0)		0.93 (0.89-0.97)	<.001	0.86 (0.82-0.90)	<.001
2017	21 702 (9.0)	3372 (10.5)		0.91 (0.87-0.96)	<.001	0.81 (0.77-0.85)	<.001
Comorbidity score^f^							
0	181 161 (74.7)	21 715 (67.4)	<.001	1 [Reference]	NA	1 [Reference]	<.001
≥1	61 447 (25.3)	10 515 (32.6)		1.46 (1.44-1.49)	<.001	1.37 (1.35-1.39)	
Neighborhood income, $^g^							
≥63 333	95 281 (39.3)	6115 (19.0)	<.001	1 [Reference]	NA	1 [Reference]	NA
50 354-63 332	59 809 (24.7)	5193 (16.1)		1.16 (1.13-1.18)	<.001	1.08 (1.06-1.10)	<.001
40 227-50 353	53 571 (22.1)	6723 (20.9)		1.26 (1.23-1.28)	<.001	1.10 (1.08-1.12)	<.001
<40 227	33 947 (14.0)	14 199 (44.1)		1.56 (1.53-1.59)	<.001	1.11 (1.08-1.13)	<.001
Insurance status^h^							
Private	124 203 (51.2)	12 655 (39.3)	<.001	1 [Reference]	NA	1 [Reference]	NA
Medicare	102 222 (42.1)	14 369 (44.6)		2.74 (2.70-2.79)	<.001	1.19 (1.17-1.22)	<.001
Medicaid	9872 (4.1)	3377 (10.5)		2.40 (2.32-2.48)	<.001	1.58 (1.53-1.63)	<.001
Uninsured	6311 (2.6)	1829 (5.7)		2.06 (1.98-2.15)	<.001	1.51 (1.45-1.57)	<.001
Histologic grade (G)^i^							
Endometrioid G1	84 288 (34.7)	6024 (18.7)	<.001	1 [Reference]	NA	1 [Reference]	NA
Endometrioid G2	53 340 (22.0)	4851 (15.1)		1.83 (1.79-1.88)	<.001	1.42 (1.38-1.45)	<.001
Endometrioid G3	23 053 (9.5)	3774 (11.7)		4.35 (4.24-4.47)	<.001	2.40 (2.34-2.47)	<.001
Endometrioid GX	29 557 (12.2)	2739 (8.5)		2.04 (1.97-2.10)	<.001	1.46 (1.41-1.51)	<.001
Mixed	13 318 (5.5)	1932 (6.0)		3.15 (3.04-3.26)	<.001	2.00 (1.93-2.07)	<.001
Serous	13 630 (5.6)	5236 (16.3)		6.59 (6.401-6.77)	<.001	2.55 (2.48-2.63)	<.001
Clear cell	3107 (1.3)	838 (2.6)		5.71 (5.44-5.99)	<.001	2.43 (2.31-2.55)	<.001
Carcinosarcoma	8955 (3.7)	3617 (11.2)		9.39 (9.12-9.67)	<.001	4.05 (3.92-4.18)	<.001
Other cell types	13 360 (5.5)	3219 (10.0)		6.50 (6.32-6.69)	<.001	3.15 (3.06-3.25)	<.001
Stage^j^							
I	180 338 (74.3)	18 759 (58.2)	<.001	1 [Reference]	NA	1 [Reference]	NA
II	14 345 (5.9)	2489 (7.7)		2.38 (2.32-2.45)	<.001	1.92 (1.86-1.97)	<.001
III	30 021 (12.4)	5583 (17.3)		4.02 (3.94-4.02)	<.001	3.37 (3.30-3.45)	<.001
IV	17 904 (7.4)	5399 (16.8)		14.39 (14.12-14.66)	<.001	7.42 (7.24-7.61)	<.001
First-line treatment^k^							
Surgery	229 139 (94.5)	28 283 (87.8)	<.001	1 [Reference]	NA	1 [Reference]	NA
No surgery	13 469 (5.6)	3947 (12.3)		7.90 (7.75-8.06)	<.001	3.05 (2.98-3.12)	<.001
Radiotherapy	68 788 (28.4)	9852 (30.6)	<.001	1 [Reference]	NA	1 [Reference]	NA
No radiotherapy	173 820 (71.7)	22 378 (69.4)		0.78 (0.77-0.79)	<.001	1.17 (1.15-1.19)	<.001
Chemotherapy	47 182 (19.4)	10 908 (33.8)	<.001	1 [Reference]	NA	1 [Reference]	NA
No chemotherapy	195 462 (80.6)	21 322 (66.2)		0.36 (0.35-0.36)	<.001	1.25 (1.22-1.27)	<.001

Abbreviations: G, grade; HR, hazard ratio; NA, not applicable.

^a^ The distribution of clinical variables in the unadjusted population were compared between non-Hispanic White and non-Hispanic Black patients with uterine cancer using the *t* test (for age) and the χ^2^ test (for all other categorical variables) with the associated *P* value.

^b^ Unadjusted HR and 95% CI were estimated for each variable from univariable Cox proportional hazards regression analysis of overall survival with the associated *P* value; HR for age indicates relative risk per 5-year increase.

^c^ Adjusted HR and 95% CI were estimated for each variable from a full multivariable Cox proportional hazards regression model including race, age, Census division, year of diagnosis, comorbidity score, neighborhood income, insurance status, histologic subtype, disease stage, and treatment with the associated *P* value; HR for age indicates relative risk per 5-year increase.

^d^ Black and White patients were defined based on the 2 self-reported variables coded by the National Cancer Database, race, and Hispanic or Spanish ethnicity. Other racial and ethnic groups were excluded.

^e^ Baseline demographic characteristics included age, Census division of residence, and year at diagnosis; patients with missing data in Census division were not included.

^f^ Comorbidity score was measured by the Charlson-Deyo score system and categorized as 0 or 1 or more. A high comorbidity score (≥1) refers to a symptomatic patient.

^g^ Neighborhood income was indicated with the median household income for each patient’s area of residence by matching the zip code against files derived from the 2016 American Community Survey data. Patients with missing neighborhood income data were excluded.

^h^ Insurance status was categorized as uninsured, Medicaid, Medicare, or private insurance. Patients with unknown insurance data were excluded.

^i^ Histologic subtype was defined using the *International Classification of Diseases for Oncology, Third Edition* and categorized as endometrioid (codes 8380, 8382, 8140, 8263, and 8570), mixed cell type (codes 8255 and 8323), serous (codes 8441, 8460, and 8461), clear cell (code 8310), carcinosarcoma (codes 8950 and 8980), or the other uterine cancer histologic subtypes, including both carcinomas and sarcomas. Endometrioid histologic subtype was further stratified by tumor grade.

^j^ This study used the *International Statistical Classification of Diseases and Related Health Problems, Tenth Revision* topography codes for corpus uteri and uterus, not otherwise specified. Stage was categorized as I, II, III, or IV using the International Federation of Gynecology and Obstetrics and the 6th and 7th editions of the American Joint Committee on Cancer criteria. In situ tumors or cancers with unknown stage data were excluded. Patients with multiple malignant neoplasms were also excluded.

^k^ Cancer treatment included assessments for surgery, radiotherapy, and chemotherapy. Surgery (yes or no) was defined by the surgical procedure performed to the primary site; radiotherapy (yes or no) was defined as the radiotherapy administered to the primary site or metastatic site as part of the first course of treatment; and chemotherapy (yes or no) was defined as the chemotherapy administered as the first course of treatment. Patients with unknown information in surgery, radiation, or chemotherapy were excluded.

### Statistical Analysis

Statistical analysis was performed in July 2022. The *t* test was used to compare age at diagnosis between Black and White patients and expressed with mean (SD) values. The χ^2^ test was used for categorical variables. The primary outcome was overall survival as recorded by the NCDB, with survival time calculated from the date of diagnosis to death or last contact, whichever occurred first. Associations between explanatory factors and overall survival were evaluated in the original cohort using Cox proportional hazards regression modeling with univariable and multivariable analyses. The Kaplan-Meier method was used to estimate 5-year survival. The inverse Kaplan-Meier method^54,55^ was applied to calculate the median follow-up time. The association of explanatory factors with survival disparities was investigated using a sequential propensity score balancing procedure deployed by Jemal et al.^51^ A logistic regression model was used to calculate the propensity of being Black conditional on the variables, with Black assigned a weight of (1 / propensity) and White assigned a weight of (1 / [1 − propensity]).^56^ To reduce the potential bias from extreme values, we used stabilized weighting.^57^ The quality of balance between Black and White patients was examined using the standardized mean difference, with a value of less than 10% considered well balanced.^58^

Inverse probability weighting based on propensity score was applied to sequentially balance the following complement of clinical covariates for Black and White patients in 7 steps. Step 1 balanced for demographic characteristics, including age, Census division, and year of diagnosis. Step 2 balanced for covariates from step 1 and comorbidity score. Step 3 balanced for covariates from step 2 and neighborhood income. Step 4 balanced for covariates from step 3 and insurance status. Step 5 balanced for covariates from step 4 and histologic subtype. Step 6 balanced for covariates from step 5 and disease stage. Step 7 balanced for covariates from step 6 and treatment with surgery (no or yes), radiotherapy (no or yes), and chemotherapy (no or yes).

The adjusted hazard ratio (HR) for risk of death was then evaluated in the propensity score–balanced Black and White patients using a weighted Cox proportional hazards regression model, with 95% CIs estimated using a robust sandwich variance.^59^ The total ERR of death for Black patients relative to White patients was calculated by subtracting 1 from the baseline-adjusted HR. The association of individual factors and the cumulative association with the survival difference between Black and White patients were calculated based on the reduction in HR after sequential adjustment for the additional covariates.^51,60^ Adjusted 5-year survival for Black and White patients was estimated using the weighted Kaplan-Meier method in the propensity score–balanced cohorts.^55^ After performing analyses among all patients with uterine cancer, the procedures and analyses were repeated, stratified by age with patients who received a diagnosis at an age younger than 65 years or 65 years or older to further investigate the importance of insurance status on racial disparity in this disease before vs after Medicare eligibility. All statistical analyses for this study were performed using SAS, version 9.4 (SAS Institute Inc). All tests were 2-sided, with *P* < .05 considered statistically significant. This study did not control for potential type I error due to multiple testing, and subset analyses were considered exploratory.

## Results

There were 274 838 patients (242 608 White patients [mean (SD) age at diagnosis, 63.5 (10.5) years] and 32 230 Black patients [mean (SD) age at diagnosis, 63.8 (10.0) years]) with stage I to IV uterine cancer who satisfied the eligibility criteria (Table 1). A total of 11.7% patients were self-described as Black, 26.2% had comorbidities, 24.5% received a diagnosis of nonendometrioid subtype, and 21.5% had stage III to IV disease beyond the uterus. Table 1 shows the clinical characteristics that varied by race. Black patients were more likely than White patients to have a comorbidity score of 1 or more (32.6% vs 25.3%; *P* < *.*001), low income (44.1% vs 14.0%; *P* < *.*001), no insurance or Medicaid insurance (16.2% vs 6.7%; *P* < *.*001), nonendometrioid histologic characteristics (46.1% vs 21.6%; *P* < *.*001), advanced disease stage (34.1% vs 19.8%; *P* < *.*001), no surgery (12.3% vs 5.6%; *P* < *.*001), and chemotherapy (33.8% vs 19.4%; *P* < *.*001), while the use of radiotherapy was modestly different between Black and White patients (eFigure 2 in Supplement 1). Treatment with hormone therapy (1.7% of Black patients vs 1.3% of White patients) or immunotherapy (0.2% of White patients vs 0.1% of White patients) was rare in both groups.

**Figure 1.**
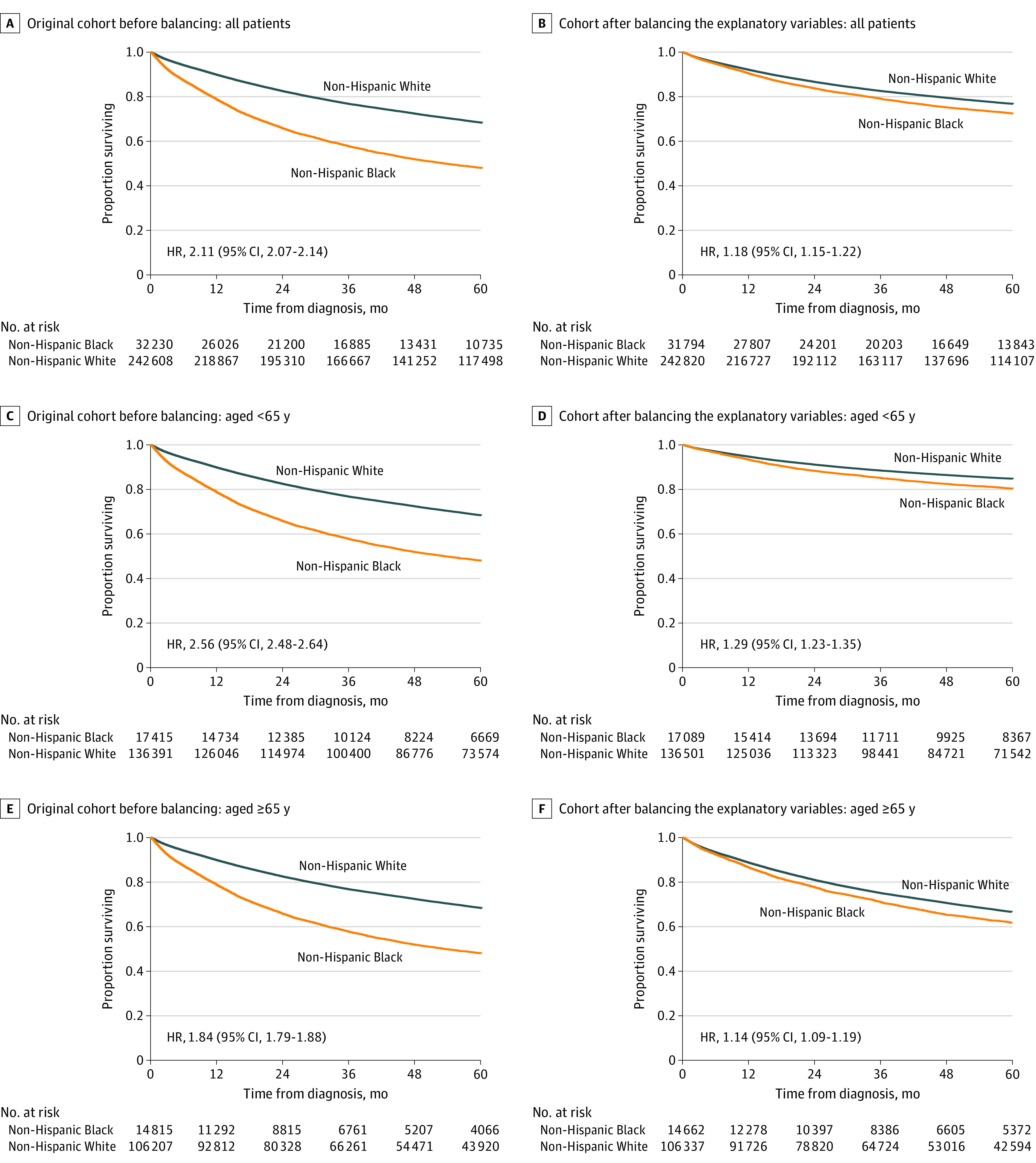
Survival Disparities Among Non-Hispanic Black and Non-Hispanic White Patients With Uterine Cancer Unadjusted and adjusted survival distributions were evaluated using the Kaplan-Meier method and compared using the log-rank test. HR indicates hazard ratio.

At the time of this analysis, 26.7% of patients had died, and the median follow-up time was 74.0 months (range, 43.5-113.8 months). Figure 1A illustrates that Black patients in the original cohort before balancing had significantly worse survival than White patients, with a 5-year survival of 58.6% for Black patients vs 78.5% for White patients. The unadjusted risk of death was 2.11-fold higher for Black vs White patients (95% CI, 2.07-2.14). The racial disparity in survival persisted for patients who received a diagnosis at an age younger than 65 years (Figure 1C) and those who received a diagnosis at 65 years or older (Figure 1E) in the original cohort before balancing. The magnitude of the survival disparity was larger for patients who received a diagnosis at younger than 65 years, with an unadjusted HR of 2.56 (95% CI, 2.48-2.64) before Medicare eligibility (Figure 1C), and 1.84 (95% CI, 1.79-1.88) after Medicare eligibility (Figure 1E). The distribution of insurance status varied significantly for Black vs White patients who received a diagnosis at younger than 65 years (eFigure 3A in Supplement 1) but was similar for those who received a diagnosis at 65 years or older (eFigure 3B in Supplement 1).

The associations of race, age, Census division, year of diagnosis, comorbidity score, neighborhood income, insurance status, histologic subtype, disease stage, and treatment with survival are also presented in Table 1. Histologic subtype, disease stage, and first-line treatment status were the dominant factors independently associated with worse survival among Black vs White patients. These univariable and multivariable survival analyses do not indicate how much each of these factors was associated with the survival disparities.

**Figure 2.**
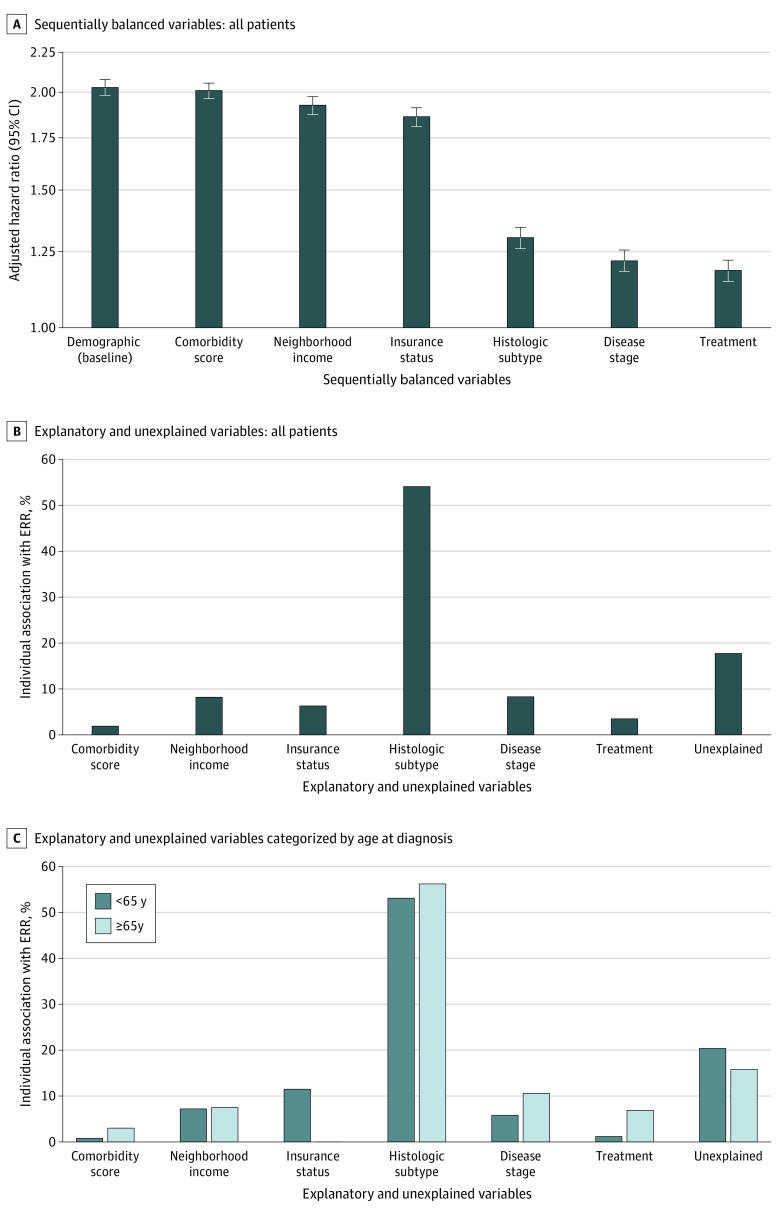
Adjusted Hazard Ratio and Excess Relative Risk (ERR) for Non-Hispanic Black vs Non-Hispanic White Patients A, Adjusted hazard ratios for risk of death for non-Hispanic Black patients compared with non-Hispanic White patients for each sequentially balanced variable. Error bars indicate 95% CIs. B, Individual associations of the explanatory and unexplained variables with the percentage ERR of death for non-Hispanic Black patients compared with non-Hispanic White patients who received a diagnosis of uterine cancer at all ages. C, Individual associations of the explanatory and unexplained variables with the percentage ERR of death for non-Hispanic Black patients compared with non-Hispanic White patients who received a diagnosis of uterine cancer at younger than 65 years vs 65 years or older.

Propensity score weighting was applied to incrementally balance each of the potential explanatory study factors, and the change of ERR was determined. Table 2 displays the distribution of characteristics among White and Black patients during each step of the sequential propensity score balancing. Each variable was determined to be well balanced after propensity score weighting, with a standardized mean difference of less than 10%. Table 3 shows that the HR for Black vs White patients in the baseline demographic model after balancing for age, Census division, and year of diagnosis was 2.03 (95% CI, 1.98-2.08) and decreased to 2.01 (95% CI, 1.96-2.06), 1.93 (95% CI, 1.87-1.98), 1.86 (95% CI, 1.81-1.91), 1.30 (95% CI, 1.26-1.34), 1.22 (95% CI, 1.18-1.26), and 1.18 (95% CI, 1.15-1.22) after further balancing for comorbidity score, neighborhood income, insurance status, histologic subtype, disease stage, and treatment, respectively (Figure 2A). These factors accounted for 82.3% of the total ERR of death for Black patients relative to White patients. The individual association with ERR was 1.9% due to comorbidity score, 8.2% due to neighborhood income, 6.3% due to insurance status, 54.1% due to histologic subtype, 8.3% due to disease stage, and 3.5% due to treatment, and 17.7% remained unexplained (Figure 2B). Table 3 also shows the cumulative association with the ERR.

**Table 2.** Distribution of Clinical Characteristics in the White vs Black Patients With Uterine Cancer by Applying the Sequential Propensity Score Balancing Procedure

Characteristic	Distribution of patient characteristics after sequential balancing, %^a^													
	Step 1		Step 2		Step 3		Step 4		Step 5		Step 6		Step 7	
	Demographic (baseline)		Comorbidity score		Neighborhood income		Insurance status		Histologic subtype		AJCC stage		Treatment	
	White	Black	White	Black	White	Black	White	Black	White	Black	White	Black	White	Black
Age at diagnosis, mean, y	63.5	63.4	63.5	63.4	63.5	63.2	63.5	63.1	63.5	63.1	63.5	63.1	63.5	63.1
Census division														
New England	6.9	6.9	6.9	6.8	6.9	6.9	6.9	6.6	6.9	6.6	6.9	6.6	6.9	6.7
Middle Atlantic	16.9	16.8	16.9	16.8	16.9	16.9	16.9	16.6	16.9	16.4	16.9	16.3	16.9	16.4
East North Central	21.1	21.2	21.1	21.2	21.1	21.2	21.1	21.4	21.1	21.1	21.1	21.1	21.1	21.2
West North Central	19.2	19.2	19.2	19.3	19.2	19.2	19.2	19.4	19.2	19.6	19.2	19.6	19.2	19.5
South Atlantic	6.9	6.9	6.9	6.9	6.9	6.9	6.9	7.0	6.9	7.1	6.9	7.1	6.9	7.2
East South Central	8.4	8.4	8.4	8.3	8.4	8.3	8.4	8.2	8.4	8.4	8.4	8.4	8.4	8.3
West South Central	6.2	6.2	6.2	6.2	6.2	6.2	6.2	6.2	6.2	6.2	6.2	6.2	6.2	6.2
Mountain	3.6	3.7	3.6	3.7	3.6	3.7	3.6	3.7	3.6	3.7	3.6	3.7	3.6	3.7
Pacific	10.8	10.8	10.8	10.9	10.8	10.8	10.8	10.9	10.8	11.0	10.8	11.0	10.8	11.0
Year of diagnosis														
2004	4.6	4.5	4.6	4.5	4.6	4.3	4.6	4.2	4.6	4.2	4.6	4.2	4.6	4.2
2005	4.9	4.9	4.9	4.9	4.9	4.8	4.9	4.7	4.9	4.9	4.9	4.9	4.9	4.9
2006	5.3	5.3	5.3	5.3	5.3	5.1	5.3	5.1	5.3	5.2	5.3	5.2	5.3	5.2
2007	5.6	5.7	5.6	5.7	5.6	5.4	5.6	5.4	5.6	5.3	5.6	5.3	5.6	5.3
2008	6.1	6.1	6.1	6.1	6.1	5.9	6.1	5.8	6.1	5.8	6.1	5.8	6.1	5.8
2009	6.3	6.3	6.3	6.4	6.3	6.3	6.3	6.3	6.4	6.2	6.4	6.2	6.4	6.2
2010	7.3	7.1	7.3	7.1	7.3	7.1	7.3	7.0	7.3	7.2	7.3	7.2	7.3	7.2
2011	7.7	7.7	7.7	7.7	7.7	7.9	7.7	7.9	7.7	7.9	7.7	7.9	7.7	7.9
2012	8.0	8.0	8.0	8.0	8.0	8.0	8.0	8.1	8.0	8.2	8.0	8.2	8.0	8.3
2013	8.4	8.5	8.4	8.5	8.4	8.4	8.4	8.5	8.4	8.6	8.4	8.6	8.4	8.6
2014	8.7	8.7	8.7	8.7	8.7	8.8	8.7	8.8	8.7	8.6	8.7	8.6	8.7	8.6
2015	8.9	8.9	8.9	8.9	8.9	9.0	8.9	9.0	8.9	8.8	8.9	8.8	8.9	8.8
2016	9.0	9.2	9.0	9.2	9.0	9.5	9.0	9.6	9.0	9.7	9.0	9.7	9.0	9.7
2017	9.1	9.2	9.1	9.3	9.1	9.4	9.1	9.5	9.1	9.4	9.1	9.4	9.1	9.5
Comorbidity score														
0	74.6	67.6	73.8	73.6	73.8	73.1	73.8	72.8	73.8	72.8	73.8	72.8	73.8	72.8
≥1	25.4	32.4	26.2	26.5	26.2	26.9	26.2	27.2	26.2	27.2	26.2	27.2	26.2	27.2
Neighborhood income, $														
≥63 333	38.9	20.9	38.9	21.0	36.9	36.7	36.9	36.4	36.7	36.5	36.9	36.5	36.9	36.5
50 354-63 332	24.5	17.1	24.5	17.2	23.7	23.6	23.6	23.6	23.6	23.3	23.6	23.3	23.6	23.2
40 227-50 353	22.3	20.2	22.3	20.1	21.9	22.1	21.9	22.1	21.9	22.3	21.9	22.4	21.9	22.4
<40 227	14.4	41.9	14.4	41.7	17.5	17.7	17.6	18.0	17.6	17.9	17.6	17.9	17.6	18.0
Insurance status														
Private	51.0	40.1	50.9	40.7	50.6	43.5	49.8	50.0	49.7	49.8	49.7	49.8	49.7	49.8
Medicare	42.3	43.0	42.4	42.4	42.5	41.1	42.4	41.4	42.4	41.6	42.4	41.5	42.4	41.4
Medicaid	4.0	11.7	4.1	11.6	4.2	10.3	4.9	5.4	4.9	5.4	4.9	5.4	4.9	5.5
Uninsured	2.6	5.3	2.7	5.5	2.7	5.1	3.0	3.2	3.0	3.2	3.0	3.2	3.0	3.3
Histologic grade (G)														
Endometrioid G1	34.7	19.3	34.7	19.2	34.6	19.6	34.5	20.0	32.9	32.7	32.8	32.8	32.8	32.7
Endometrioid G2	22.0	15.4	22.0	15.4	22.1	15.5	22.1	15.6	21.2	22.1	21.2	22.2	21.2	22.2
Endometrioid G3	9.5	11.9	9.5	11.9	9.5	11.6	9.5	11.5	9.8	9.6	9.8	9.6	9.8	9.7
Endometrioid GX	12.2	8.1	12.2	8.1	12.2	8.1	12.2	8.1	11.8	11.3	11.7	11.1	11.7	11.1
Mixed	5.5	6.1	5.5	6.0	5.5	6.0	5.5	6.0	5.6	5.8	5.6	5.7	5.6	5.7
Serous	5.6	15.9	5.6	16.0	5.6	16.0	5.6	16.0	6.8	6.7	6.8	6.7	6.8	6.7
Clear cell	1.3	2.5	1.3	2.5	1.3	2.4	1.3	2.3	1.4	1.3	1.4	1.3	1.4	1.3
Carcinosarcoma	3.7	11.2	3.7	11.2	3.7	11.1	3.7	10.9	4.6	4.6	4.6	4.6	4.6	4.6
Other cell types	5.5	9.6	5.5	9.7	5.5	9.7	5.5	9.5	6.0	5.9	6.0	5.9	6.1	6.0
Stage														
I	74.3	58.2	74.3	58.1	74.2	59.1	74.1	60.0	72.9	69.9	72.4	72.9	72.4	72.9
II	5.9	7.8	5.9	7.7	6.0	7.4	6.0	7.3	6.0	6.7	6.1	6.0	6.1	6.0
III	12.4	17.3	12.4	17.3	12.4	17.0	12.4	16.8	12.9	12.5	12.9	12.3	12.9	12.3
IV	7.4	16.8	7.4	16.9	7.4	16.5	7.5	16.0	8.1	10.9	8.6	8.9	8.6	8.8
First-line treatment														
Surgery	94.4	87.8	94.8	87.9	94.4	88.6	94.3	89.1	94.1	89.8	94.0	90.7	93.6	93.1
No surgery	5.6	12.2	5.6	12.2	5.6	11.4	5.7	11.0	5.9	10.2	6.0	9.3	6.4	6.9
Radiotherapy	28.3	31.0	28.2	31.0	28.3	30.6	28.3	30.5	28.7	26.9	28.7	26.7	28.6	27.5
No radiotherapy	71.8	69.1	71.8	69.1	71.7	69.5	71.7	69.5	71.3	73.1	71.3	73.3	71.4	72.5
Chemotherapy	19.5	33.6	19.4	33.8	19.5	33.5	19.5	33.2	20.9	21.9	21.1	20.8	21.1	20.9
No chemotherapy	80.5	66.4	80.6	66.2	80.5	66.5	80.5	66.8	79.1	78.1	78.9	79.2	78.9	79.1

Abbreviations: AJCC, American Joint Committee on Cancer; G, grade.

^a^ A propensity score balancing procedure was applied in 7 steps to the original study population composed of 242 608 White and 32 230 Black patients. Step 1 balanced demographic characteristics, including age at diagnosis, Census division, and year of diagnosis. Step 2 extended balancing of demographic characteristics to include comorbidity score. Step 3 extended balancing of demographic and comorbidity characteristics to include neighborhood income. Step 4 extended balancing of demographic characteristics, comorbidity score, and neighborhood income to include insurance status. Step 5 extended balancing of demographic characteristics, comorbidity score, neighborhood income, and insurance status to include histologic subtype. Step 6 extended balancing of demographic characteristics, comorbidity score, neighborhood income, insurance status, and histologic subtype to include stage. Step 7 extended balancing of demographic characteristics, comorbidity score, neighborhood income, insurance status, histologic subtype, and disease stage to include treatment with surgery (no vs yes), radiotherapy (no vs yes), and chemotherapy (no vs yes).

**Table 3.** Factors Associated With Risk of Death, ERR of Death, Individual Association With ERR, and Cumulative Association With ERR Between Black and White Patients With Uterine Cancer

Patient group	HR (95% CI)^a^	ERR^b^	Individual association with ERR, %^c^	Cumulative association with ERR, %^c^
**All patients (aged 40-90 y)**				
Sequentially balanced variables^d^				
Step 1: demographic (baseline)	2.03 (1.98-2.08)	1.03	NA	NA
Step 2: adding comorbidity score	2.01 (1.96-2.06)	1.01	1.9	1.9
Step 3: adding neighborhood income	1.93 (1.87-1.98)	0.93	8.2	10.1
Step 4: adding insurance status	1.86 (1.81-1.91)	0.86	6.3	16.4
Step 5: adding histologic subtype	1.30 (1.26-1.34)	0.30	54.1	70.5
Step 6: adding stage	1.22 (1.18-1.26)	0.22	8.3	78.8
Step 7: adding treatment	1.18 (1.15-1.22)	0.18	3.5	82.3
Unexplained	NA	NA	17.7	100
**Aged <65 y**				
Sequentially balanced variables^d^				
Step 1: demographic (baseline)	2.43 (2.34-2.52)	1.43	NA	NA
Step 2: adding comorbidity score	2.42 (2.33-2.50)	1.42	0.8	0.8
Step 3: adding neighborhood income	2.31 (2.22-2.41)	1.31	7.2	8.0
Step 4: adding insurance status	2.15 (2.06-2.24)	1.15	11.5	19.5
Step 5: adding histologic subtype	1.39 (1.33-1.46)	0.39	53.1	72.6
Step 6: adding stage	1.31 (1.25-1.37)	0.31	5.8	78.4
Step 7: adding treatment	1.29 (1.23-1.35)	0.29	1.2	79.6
Unexplained	NA	NA	20.4	100
**Aged ≥65 y**				
Sequentially balanced variables^d^				
Step 1: demographic (baseline)	1.87 (1.81-1.93)	0.87	NA	NA
Step 2: adding comorbidity score	1.84 (1.79-1.90)	0.84	3.0	3.0
Step 3: adding neighborhood income	1.78 (1.71-1.85)	0.78	7.5	10.5
Step 4: adding insurance status	1.78 (1.71-1.85)	0.78	0.0	10.5
Step 5: adding histologic subtype	1.29 (1.23-1.35)	0.29	56.2	66.7
Step 6: adding stage	1.20 (1.14-1.25)	0.20	10.6	77.3
Step 7: adding treatment	1.14 (1.09-1.19)	0.14	6.9	84.2
Unexplained	NA	NA	15.8	100

Abbreviations: ERR, excess relative risk; HR, hazard ratio; NA, not applicable.

^a^ Weighted Cox proportional hazards regression modeling was performed to estimate the HR and the 95% CI in the baseline model at birth with adjustments for age, Census division, and year of diagnosis, and then after each sequential balancing step.

^b^ The total ERR of death in Black patients relative to White patients was calculated by subtracting 1 from the baseline-adjusted HR.

^c^ The contribution of the individual association with the ERR and the cumulative association with the ERR between Black and White patients was calculated based on the reduction in HR after sequential adjustment for the additional covariates. The proportion of total ERR explained by each factor derived as ([HR_b_ − HR_a_] / [1 − HR_baseline_]) × 100%, where HR_b_ indicates the HR estimate before balance of the corresponding factor, HR_a_ indicates the HR estimate after balance of the corresponding factor, and HR_baseline_ indicates the HR from baseline model.

^d^ Propensity score analysis was applied to sequentially balance the following complement of clinical covariates in Black and White patients in a 7-step process. Step 1 balanced demographic characteristics, including age at diagnosis, Census division, and year of diagnosis at birth. Steps 2 through 4 balanced factors prior to diagnosis. Step 2 extended balancing of demographic factors to include comorbidity score. Step 3 extended balancing of demographic and comorbidity factors to include neighborhood income. Step 4 extended balancing of demographic characteristics, comorbidity score, and neighborhood income to include insurance status. Steps 5 and 6 balanced factors at diagnosis. Step 5 extended balancing of demographic characteristics, comorbidity score, neighborhood income, and insurance status to include histologic subtype. Step 6 extended balancing of demographic characteristics, comorbidity score, neighborhood income, insurance status, and histologic subtype to include disease stage. Step 7 extended balancing of demographic characteristics, comorbidity score, neighborhood income, insurance status, histologic subtype, and disease stage to include factors regarding first-line treatment with surgery (no vs yes), radiotherapy (no vs yes), and chemotherapy (no vs yes).

Analyses were then performed for subsets of patients who received a diagnosis of uterine cancer before and after Medicare eligibility at 65 years of age to further investigate the association of modifiable factors, such as insurance status, with survival (Table 3). There were 17 415 Black and 136 391 White patients who received a diagnosis at younger than 65 years, whereas 14 815 Black and 106 207 White patients received a diagnosis at 65 years or older. Before balancing, 25.3% of Black patients and 10.8% of White patients younger than 65 years were uninsured or had Medicaid insurance at diagnosis. The proportion with no insurance or Medicaid insurance decreased to 5.4% of Black patients and 1.4% of White patients who received a diagnosis at 65 years or older. The distribution of clinical characteristics after applying the sequential propensity score balancing procedure among Black vs White patients who received a diagnosis of uterine cancer at younger than 65 years or 65 years or older are shown in eTable 1 and eTable 2 in Supplement 1, respectively. Each variable, including insurance status, was confirmed to be well balanced between Black and White patients (standardized mean difference, <10%).

For patients who received a diagnosis at younger than 65 years, the HR for death for Black vs White patients was 2.43 (95% CI, 2.34-2.52) in the baseline demographic-adjusted model, which was reduced to 1.29 (95% CI, 1.23-1.35) after balancing other factors (Table 3). Comorbidity score, neighborhood income, insurance status, histologic subtype, disease stage, treatment, and unexplained factors accounted for 0.8%, 7.2%, 11.5%, 53.1%, 5.8%, 1.2%, and 20.4%, respectively, of the total ERR for younger Black vs White patients. For patients 65 years or older at diagnosis, the HR for death for Black vs White patients was 1.87 (95% CI, 1.81-1.93) in the baseline demographic-adjusted model, which was reduced to 1.14 (95% CI, 1.09-1.19) after balancing other factors. Comorbidity score, neighborhood income, insurance status, histologic subtype, disease stage, treatment, and unexplained factors accounted for 3.0%, 7.5%, 0.0%, 56.2%, 10.6%, 6.9% and 15.8%, respectively, of the total ERR for Medicare-eligible patients. Insurance status was associated with 11.5% of the survival difference between Black and White among patients younger than 65 years and was not associated with racial disparity for patients 65 years or older (Figure 2C).

## Discussion

Data from the NCDB were used to investigate factors associated with the health disparities in survival between Black and White patients with uterine cancer before and after the age of Medicare eligibility. Histologic subtype was the dominant factor associated with differences in survival by race in this investigation, accounting for 53.1% of the total ERR for patients younger than 65 years and 56.2% of the total ERR for those 65 years or older. However, insurance status was the largest modifiable factor, accounting for 11.5% of the ERR for women younger than 65 years. These findings merit consideration by policy makers, researchers, and clinicians in allocating resources and setting research priorities.

The study by Jemal et al^51^ of nonelderly patients with breast cancer (<65 years) demonstrated that insurance status accounted for 37% of the total ERR and reinforced the benefits associated with national screening programs. In contrast, our study found that insurance status was associated with only 11.5% of the total ERR for women younger than 65 years and had no association for women 65 years or older. However, insurance status still represented the largest modifiable risk factor for patients younger than 65 years in our study. The differences in insurance status before and after 65 years of age are associated primarily with Medicare eligibility and enrollment. Barrington et al^61^ found that Medicaid expansion after the passage of the Patient Protection and Affordable Care Act increased insurance coverage, was associated with earlier disease stages at diagnosis, and improved survival among women 53 to 57 years of age with uterine cancer. Furthermore, the association of insurance status with survival disparities between Black and White patients held up in a propensity score balancing analysis among the subset of patients with the most common histologic subtype of uterine cancer, low-grade endometrioid adenocarcinoma, and persisted among patients with higher-risk histologic subtypes.^62^ By using propensity score balancing, we aimed to control for variations in treatment among the other covariates and demonstrated that, regardless of histologic subtype, patient insurance status remained independently associated with survival. However, our analysis does not account for differences in patient-specific treatment regimens, timing of treatments, or completion of treatments, and these factors may be associated at least in part with the disparities in survival. In addition, various insurance providers have different supportive care services, which may be associated with variations in the completion of treatments and outcomes. In addition, there are many confounding factors in the association of insurance status with survival. Medicaid expansion is state based and has strong regional trends that further complicate its association with survival. Future investigations will need to reexamine the association of Medicaid expansion with uterine cancer prevention, diagnosis, treatment, survival, and health equity. Although insurance status was the largest modifiable factor associated with ERR for younger women, we know that even among populations with equal access to care, there continue to be health disparities in survival for Black vs White patients with uterine cancer.^15^

Another significant finding of our study was the percentage associated with ERR in survival for histologic subtype. Other studies have shown the differences between histologic subtype, disease stage, and grade for White vs Black patients.^6,7,8,9,10,11,12,13,14,15,16,24,26,27,28,29,30,31,32,33,34,35,36,37,38,39,63,64^ However, these studies did not analyze the percentage associated with ERR while including demographic characteristics, comorbidity score, insurance status, neighborhood income, disease stage, histologic subtype, and treatment with surgery, radiotherapy, and/or chemotherapy. We hypothesize that the association of molecular factors with the histologic subtypes of uterine cancer may explain 53% to 56% of the ERR differences between Black and White patients in this study. Some histologic differences might be associated with age due to unopposed estrogen or obesity in younger patients with uterine cancer.^4,12,65,66^ In addition, a growing body of evidence has also documented inherited and somatic alterations in suppressors, oncogenes, mismatch repair, immune checkpoints, and molecular subtypes among Black and White patients with uterine cancer associated with aggressive histologic subtypes that merit further study using molecular profiling platforms, predictive algorithms, digital pathology, natural language processing, and deep machine learning.^12,20,21,22,23,41,42,43,44,45,46,47,48,49,50,67,68^ Our study also demonstrated that 20.4% and 15.8% of the survival disparities between Black and White patients remained unexplained among women who received a diagnosis at younger than 65 years vs 65 years or older, respectively. We need to better understand the association of ancestry, environment, exposures, lifestyle, and structural determinants of health with disease susceptibility, severity, and treatment efficacy to achieve more equitable outcomes, including among those who develop aggressive and deadly uterine cancers, such as high-risk endometrioid cancer, uterine serous cancer, and carcinosarcoma.^69,70^

### Limitations

We recognize limitations in our study associated with the use of clinical cancer registry data from CoC-accredited hospitals with a limited set of variables and the multifactorial nature of survival disparities. The lack of available data on social determinants of health (eg, barriers to care; food, education, housing, and economic insecurities; access to care; environmental factors; or social community context), cause of death, recurrence or progression events, cancer agent details, genetic ancestry, and molecular annotation for uterine cancer cases in the NCDB is also a limitation. These factors are likely associated with some of the health disparities in uterine cancer survival and deserve research attention. The retrospective nature of database research, the lack of patient-level income and education data, the exclusion of cases with missing first-line treatment or survival data, and the inability to centrally review data with source documents are other limitations. Neighborhood educational level was excluded from our analysis due to its strong correlation with neighborhood income in preliminary analyses and the increased availability of income data compared with education data in the NCDB. Because the NCDB includes data only from CoC-accredited hospitals, it was not possible for our study to evaluate racial disparities in survival in uterine cancer or the factors associated with the differences in nonaccredited facilities. Another limitation of our study is the chosen order of sequential balancing. We incorporated variables longitudinally, starting with patient factors from birth through life, diagnosis, and first-line treatment as used by Jemal et al.^51^ eTable 3 in Supplement 1 displays point estimates from a sensitivity analysis after changing the order of the sequential balancing, moving histologic subtype and stage at diagnosis up to steps 2 and 3 in the 7-step procedure. The survival disparities between Black and White patients who received a diagnosis at younger than 65 years associated with histologic subtype increased from 53.1% to 60.3%, and the survival disparity associated with insurance status decreased from 11.5% to 3.8% (Table 3; eTable 3 in Supplement 1). We also acknowledge the complex heterogeneity and inadequacies of the common racial and ethnic categorizations used in this study. White and Black are highly simplified classifications for complex variations with intersections between ancestry (including genealogy, admixture, and exposures) and sociocultural factors (including identity, relationships, expression, risk, and structural determinants).^20,21,22,71^ This simplification of classifications will inevitably lead to overgeneralization and false conclusions, which we caution against. Although the application of propensity score balancing provides more accurate estimates compared with using conventional multivariable Cox proportional hazards regression for observational studies,^72^ it was not able to correct for the bias associated with the variables not collected by the NCDB. This study focused on health disparities between Black and White patients, excluding other groups, which merit attention and study.

## Conclusions

The results of this cohort study raise awareness of the survival disparities between Black and White patients with uterine cancer, provide insight into some of the explanatory factors, and highlight the persistent gaps in knowledge and the opportunities for further study. Insurance status represented an attractive modifiable factor for us to study, given how well it explained racial survival disparities among nonelderly women with breast cancer,^51^ but its association with survival among patients with uterine cancer was smaller due to the dominant role played by histologic subtype in this disease. These data also highlight the need to further study the survival disparities between Black and White patients with uterine cancer associated with histologic subtype and unexplained factors, including ancestry admixture, molecular alterations, barriers to care, social determinants of health, and public policy, to achieve equity and improved outcomes. Racial disparities for each uterine cancer histologic subtype should be also investigated.

## Notes

Supplement 1. **eTable 1.** The Distribution of Clinical Characteristics in the Non-Hispanic White (NHW) versus Non-Hispanic Black (NHB) Patients Diagnosed With Uterine Cancer at <65 Years Old by Applying the Sequential Propensity Score Balancing Procedure **eTable 2.** The Distribution of Clinical Characteristics in the Non-Hispanic White (NHW) vs Non-Hispanic Black (NHB) Patients Diagnosed With Uterine Cancer at ≥65 Years Old by Applying the Sequential Propensity Score Balancing Procedure **eTable 3.** Sensitivity Analysis of Factors Associated With Risk of Death, Excess Relative Risk of Death, and Both the Percent Individual Contribution (IC) and the Cumulative Contribution to the Survival Disparities Between Non-Hispanic Black (NHB) and Non-Hispanic White (NHW) Patients With Uterine Cancer Diagnosed at All Ages, the Subset Diagnosed at <65 Years Old or the Subset Diagnosed at ≥65 Years Old by Applying a Sequential Propensity Score Balancing Procedure **eFigure 1.** Schema for Selection of Study Population **eFigure 2**. The Distribution of Clinical Characteristics Associated With Poor Prognosis in Non-Hispanic White (NHW, Blue) vs Non-Hispanic Black (NHB, Red) Patients With Uterine Cancer **eFigure 3.** The distribution of Insurance Status in Non-Hispanic White (NHW, Blue) vs Non-Hispanic Black (NHB, Red) Patients With Uterine Cancer Stratified by Age at Diagnosis

Supplement 2.
Data Sharing Statement
