# Supplementary material for: Factors Associated With Survival Disparities Between Non-Hispanic Black and White Patients With Uterine Cancer
Source: JAMA Netw Open. 2023 Apr 17;6(4):e238437. doi: 10.1001/jamanetworkopen.2023.8437 (PMC10111180; doi:10.1001/jamanetworkopen.2023.8437)
Supplement: Supplement 1. — eTable 1. The Distribution of Clinical Characteristics in the Non-Hispanic White (NHW) versus Non-Hispanic Black (NHB) Patients Diagnosed With Uterine Cancer at <65 Years Old by Applying the Sequential Propensity Score Balancing Procedure eTable 2. The Distribution of Clinical Characteristics in the Non-Hispanic White (NHW) vs Non-Hispanic Black (NHB) Patients Diagnosed With Uterine Cancer at ≥65 Years Old by Applying the Sequential Propensity Score Balancing Procedure eTable 3. Sensitivity Analysis of Factors Associated With Risk of Death, Excess Relative Risk of Death, and Both the Percent Individual Contribution (IC) and the Cumulative Contribution to the Survival Disparities Between Non-Hispanic Black (NHB) and Non-Hispanic White (NHW) Patients With Uterine Cancer Diagnosed at All Ages, the Subset Diagnosed at <65 Years Old or the Subset Diagnosed at ≥65 Years Old by Applying a Sequential Propensity Score Balancing Procedure eFigure 1. Schema for Selection of Study Population eFigure 2. The Distribution of Clinical Characteristics Associated With Poor Prognosis in Non-Hispanic White (NHW, Blue) vs Non-Hispanic Black (NHB, Red) Patients With Uterine Cancer eFigure 3. The distribution of Insurance Status in Non-Hispanic White (NHW, Blue) vs Non-Hispanic Black (NHB, Red) Patients With Uterine Cancer Stratified by Age at Diagnosis [file jamanetwopen-e238437-s001.pdf]

Supplementary Online Content

Kucera CW, Tian C, Tarney CM, et al. Factors associated with survival disparities between non-Hispanic Black and White patients with uterine cancer. *JAMA Netw Open.* 2023;6(4):e238437. doi:10.1001/jamanetworkopen.2023.8437

eTable 1. The Distribution of Clinical Characteristics in the Non-Hispanic White (NHW) versus Non-Hispanic Black (NHB) Patients Diagnosed With Uterine Cancer at <65 Years Old by Applying the Sequential Propensity Score Balancing Procedure

eTable 2. The Distribution of Clinical Characteristics in the Non-Hispanic White (NHW) vs Non-Hispanic Black (NHB) Patients Diagnosed With Uterine Cancer at ≥65 Years Old by Applying the Sequential Propensity Score Balancing Procedure

eTable 3. Sensitivity Analysis of Factors Associated With Risk of Death, Excess Relative Risk of Death, and Both the Percent Individual Contribution (IC) and the Cumulative Contribution to the Survival Disparities Between Non-Hispanic Black (NHB) and Non-Hispanic White (NHW) Patients With Uterine Cancer Diagnosed at All Ages, the Subset Diagnosed at <65 Years Old or the Subset Diagnosed at ≥65 Years Old by Applying a Sequential Propensity Score Balancing Procedure

eFigure 1. Schema for Selection of Study Population

eFigure 2. The Distribution of Clinical Characteristics Associated With Poor Prognosis in Non-Hispanic White (NHW, Blue) vs Non-Hispanic Black (NHB, Red) Patients With Uterine Cancer

eFigure 3. The distribution of Insurance Status in Non-Hispanic White (NHW, Blue) vs Non-Hispanic Black (NHB, Red) Patients With Uterine Cancer Stratified by Age at Diagnosis

This supplementary material has been provided by the authors to give readers additional information about their work.

© 2023 Kucera CW et al. *JAMA Network Open.*

eTable 1. The Distribution of Clinical Characteristics in the Non-Hispanic White (NHW) versus Non-Hispanic Black (NHB) Patients Diagnosed With Uterine Cancer at <65 Years Old by Applying the Sequential Propensity Score Balancing Procedure

	Distribution of Patient Characteristics after Sequential Balancing (%)[1]													
	Step 1.		Step 2.		Step 3.		Step 4.		Step 5.		Step 6.		Step 7.	
**Age <65 Years Old**	Demographic (Baseline)		Comorbidity Score		Neighborhood Income		Insurance Status		Histology		Stage		Treatment	
	NHW	NHB	NHW	NHB	NHW	NHB	NHW	NHB	NHW	NHB	NHW	NHB	NHW	NHB
**Age at Diagnosis**														
[Mean] in Years	[56.0]	[55.9]	[56.1]	[55.9]	[56.1]	[55.9]	[56.1]	[55.8]	[56.1]	[55.8]	[56.1]	[55.8]	[56.1]	[55.8]
**Census Division**														
New England	6.9	6.9	6.9	6.8	6.9	6.9	6.9	6.7	6.9	6.5	6.9	6.5	6.9	6.6
Middle Atlantic	16.4	16.2	16.4	16.2	16.4	16.3	16.4	16.1	16.4	15.9	16.4	15.9	16.4	15.9
East North Central	20.8	20.9	20.8	20.9	20.8	20.9	20.8	21.2	20.8	21.0	20.8	21.1	20.8	21.2
West North Central	19.6	19.6	19.6	19.7	19.6	19.6	19.6	19.9	19.5	19.9	19.5	19.9	19.5	19.8
South Atlantic	7.1	7.1	7.1	7.1	7.1	7.1	7.1	7.3	7.1	7.5	7.1	7.4	7.1	7.5
East South Central	8.4	8.4	8.4	8.3	8.4	8.3	8.4	8.1	8.4	8.5	8.4	8.5	8.4	8.4
West South Central	6.3	6.3	6.3	6.3	6.3	6.3	6.3	6.2	6.3	6.3	6.3	6.3	6.3	6.3
Mountain	3.8	3.9	3.8	3.9	3.8	3.9	3.8	3.9	3.8	3.9	3.8	3.9	3.8	3.9
Pacific	10.8	10.8	10.8	10.9	10.8	10.8	10.8	10.6	10.8	10.5	10.8	10.5	10.8	10.5
**Year of Diagnosis**														
2004	4.6	4.5	4.6	4.4	4.6	4.2	4.6	4.1	4.6	4.2	4.6	4.2	4.6	4.2
2005	4.9	4.9	4.9	4.9	4.9	4.7	4.9	4.7	5.0	4.8	5.0	4.8	5.0	4.8
2006	5.5	5.5	5.5	5.5	5.5	5.3	5.5	5.2	5.5	5.3	5.5	5.3	5.5	5.3
2007	5.8	6.0	5.8	6.0	5.8	5.7	5.8	5.7	5.8	5.7	5.8	5.7	5.8	5.6
2008	6.3	6.4	6.3	6.4	6.3	6.3	6.3	6.2	6.3	6.1	6.3	6.2	6.3	6.2
2009	6.5	6.4	6.5	6.4	6.5	6.3	6.5	6.2	6.5	6.2	6.5	6.3	6.5	6.3
2010	7.6	7.5	7.6	7.5	7.6	7.4	7.6	7.2	7.6	7.4	7.6	7.4	7.6	7.3
2011	7.9	7.8	7.9	7.8	7.9	8.1	7.9	8.1	7.9	8.1	7.9	8.1	7.9	8.2
2012	8.0	8.0	8.0	8.0	8.0	8.0	8.0	8.0	8.0	8.1	8.0	8.1	8.0	8.1
2013	8.4	8.4	8.4	8.4	8.4	8.5	8.4	8.6	8.4	8.7	8.4	8.7	8.4	8.7
2014	8.7	8.6	8.7	8.6	8.7	8.8	8.7	8.8	8.7	8.5	8.7	8.5	8.7	8.5
2015	8.7	8.6	8.7	8.7	8.7	8.8	8.7	8.8	8.7	8.6	8.7	8.6	8.7	8.6
2016	8.7	8.8	8.7	8.8	8.7	9.1	8.7	9.2	8.7	9.3	8.7	9.3	8.7	9.3
2017	8.5	8.7	8.5	8.7	8.5	9.0	8.5	9.1	8.5	9.1	8.5	9.0	8.5	9.0
**Comorbidity Score**														
Score 0	76.6	71.3	76.0	75.6	76.0	75.1	76.0	74.5	76.0	74.7	76.0	74.6	76.0	74.6

© 2023 Kucera CW et al. *JAMA Network Open.*

Score ≥ 1	23.4	28.8	24.0	24.4	24.0	24.9	24.0	25.5	24.0	25.4	24.0	25.4	24.0	25.4
**Neighbor Income**														
≥$63,333	39.3	20.8	39.3	21.0	37.4	37.1	37.3	36.5	37.3	36.6	37.3	36.5	37.3	36.5
$50,354 - $63,332	24.4	17.9	24.4	18.0	23.7	23.6	23.6	23.6	23.6	23.2	23.6	23.3	23.6	23.2
$40,227 - $50,353	21.9	20.9	21.9	20.8	21.8	21.9	21.8	22.2	21.8	22.4	21.8	22.4	21.8	22.5
<$40,227	14.4	40.4	14.4	40.2	17.2	17.4	17.3	17.8	17.3	17.8	17.3	17.8	17.3	17.8
**Insurance Status**														
Private	80.5	59.5	80.4	59.9	80.0	63.8	78.2	76.7	78.2	76.7	78.2	76.6	78.2	76.6
Medicare	8.7	14.6	8.7	14.2	8.9	13.4	9.3	10.0	9.3	10.0	9.3	10.1	9.3	10.0
Medicaid	6.4	17.4	6.5	17.2	6.7	15.0	7.6	8.2	7.6	8.2	7.6	8.2	7.6	8.2
Uninsured	4.4	8.6	4.4	8.7	4.5	7.9	4.9	5.1	4.9	5.1	4.9	5.1	4.9	5.2
**Histology / Grade (G)**														
Endometrioid G1	39.8	24.6	39.7	24.5	39.6	25.0	39.5	25.6	38.0	37.3	37.9	37.4	37.9	37.3
Endometrioid G2	22.0	17.1	22.0	17.0	22.1	17.3	22.1	17.4	21.4	22.6	21.4	22.7	21.4	22.7
Endometrioid G3	8.3	10.6	8.3	10.6	8.3	10.4	8.4	10.1	8.6	8.5	8.6	8.5	8.6	8.5
Endometrioid Gx	12.7	8.6	12.7	8.6	12.7	8.6	12.7	8.6	12.2	11.8	12.2	11.7	12.2	11.7
Mixed	5.0	5.6	5.0	5.6	5.0	5.6	5.0	5.6	5.1	5.2	5.0	5.2	5.1	5.2
Serous	3.2	12.0	3.2	12.0	3.2	11.8	3.2	11.8	4.2	4.2	4.3	4.2	4.3	4.2
Clear Cell	0.8	1.5	0.8	1.6	0.8	1.5	0.8	1.5	0.9	0.9	0.9	0.9	0.9	0.9
Carcinosarcoma	2.6	9.0	2.6	9.0	2.6	8.9	2.6	8.7	3.3	3.4	3.3	3.4	3.3	3.4
Other Cell Types	5.8	11.0	5.8	11.1	5.8	11.0	5.8	10.6	6.4	6.2	6.4	6.2	6.4	6.2
**Stage**														
I	77.3	63.1	77.3	62.9	77.2	63.7	77.0	65.0	75.9	74.0	75.6	75.8	75.6	75.8
II	5.3	6.9	5.3	6.9	5.3	6.5	5.4	6.4	5.4	6.0	5.5	5.3	5.5	5.4
III	11.4	15.2	11.4	15.3	11.4	15.2	11.5	14.7	11.9	10.9	11.8	11.2	11.8	11.2
IV	6.1	14.8	6.1	14.9	6.1	14.7	6.2	13.9	6.8	9.1	7.2	7.6	7.2	7.6
**Treatment**														
Surgical Treatment	96.0	90.8	96.0	90.8	95.9	91.4	95.8	92.2	95.6	93.1	95.5	93.6	95.4	94.6
No Surgery	4.0	9.2	4.0	9.2	4.1	8.6	4.2	7.8	4.4	7.0	4.5	6.4	4.6	5.4
Radiation Therapy	25.4	28.5	25.3	28.5	25.4	28.0	24.8	27.9	25.9	24.1	25.9	23.9	25.8	25.2
No Radiation	74.7	71.5	74.7	71.5	74.6	72.0	75.2	72.2	74.1	75.9	74.1	76.1	74.2	74.8
Chemotherapy	18.6	32.4	18.5	32.7	18.6	32.1	18.6	31.6	20.0	20.8	20.1	20.1	20.2	19.8
No Chemotherapy	81.5	67.6	82.5	67.3	81.4	67.9	81.4	68.4	80.0	79.2	79.9	79.9	79.8	80.2

[1] A propensity score balancing procedure was applied in seven steps to the original study population diagnosed at <65 years composed of 136,391 NHW and 17,415 NHB patients. **Step 1** balanced demographics, including age at diagnosis, census division, and year of diagnosis. **Step 2** extended balancing of demographic factors to include comorbidity score. **Steps 3** extended balancing of demographic and comorbidity factors to include neighborhood income.

© 2023 Kucera CW et al. *JAMA Network Open.*

Step 4 extended balancing of demographics, comorbidity score and neighborhood income to include insurance status. **Step 5** extended balancing of demographics, comorbidity score, neighborhood income, and insurance status to include histology. **Step 6** extended balancing of demographics, comorbidity score, neighborhood income, insurance status and histology to include stage. **Step 7** extended balancing of demographics, comorbidity score, neighborhood income, insurance status, histology, and stage to include treatment with surgery (no versus yes), radiation (no versus yes), and chemotherapy (no versus yes).

© 2023 Kucera CW et al. *JAMA Network Open.*

eTable 2. The Distribution of Clinical Characteristics in the Non-Hispanic White (NHW) vs Non-Hispanic Black (NHB) Patients Diagnosed With Uterine Cancer at ≥65 Years Old by Applying the Sequential Propensity Score Balancing Procedure

	Distribution in Each Adjusted Population after Sequential Balancing (%)[1]													
	Step 1.		Step 2.		Step 3.		Step 4.		Step 5.		Step 6.		Step 7.	
**Age ≥65 Years Old**	Demographic (Baseline)		Comorbidity Score		Neighborhood Income		Insurance Status		Histology		Stage		Treatment	
	NHW	NHB	NHW	NHB	NHW	NHB	NHW	NHB	NHW	NHB	NHW	NHB	NHW	NHB
**Age at Diagnosis**														
[Mean] in Years	[73.0]	[73.0]	[73.0]	[73.0]	[73.0]	[72.8]	[73.0]	[72.8]	[73.0]	[73.0]	[73.0]	[73.0]	[73.0]	[73.0]
**Census Division**														
New England	6.9	6.8	6.9	6.7	6.9	6.8	6.9	6.6	6.9	6.9	6.9	6.9	6.9	7.1
Middle Atlantic	17.6	17.5	17.6	17.5	17.6	17.6	17.6	17.6	17.6	17.5	17.6	17.4	17.6	17.5
East North Central	21.5	21.6	21.5	21.5	21.5	21.6	21.5	21.8	21.5	21.4	21.5	21.4	21.5	21.4
West North Central	18.7	18.8	18.7	19.0	18.7	18.7	18.7	18.6	18.7	18.7	18.7	19.0	18.7	18.8
South Atlantic	6.6	6.6	6.6	6.7	6.6	6.7	6.6	6.8	6.6	6.9	6.6	6.9	6.6	6.9
East South Central	8.4	8.4	8.4	8.1	8.4	8.1	8.4	8.0	8.4	7.8	8.4	7.6	8.4	7.5
West South Central	6.1	6.1	6.1	6.1	6.1	6.1	6.1	6.2	6.1	6.0	6.1	6.0	6.1	6.0
Mountain	3.4	3.5	3.4	3.5	3.4	3.6	3.4	3.6	3.4	3.5	3.4	3.5	3.4	3.5
Pacific	10.9	10.8	10.9	10.9	10.9	10.9	10.9	10.9	10.9	11.3	10.9	11.3	10.9	11.3
**Year of Diagnosis**														
2004	4.7	4.6	4.7	4.6	4.7	4.4	4.7	4.3	4.7	4.4	4.7	4.3	4.7	4.4
2005	4.9	4.8	4.9	4.8	4.9	4.7	4.9	4.7	4.9	5.0	4.9	5.0	4.9	4.9
2006	5.0	4.9	5.0	4.9	5.0	4.9	5.0	4.9	5.0	5.1	5.0	5.1	5.0	5.0
2007	5.4	5.4	5.4	5.4	5.4	5.2	5.4	5.2	5.4	4.9	5.4	4.9	5.4	4.9
2008	5.7	5.7	5.7	5.7	5.7	5.5	5.7	5.5	5.7	5.4	5.7	5.4	5.7	5.3
2009	6.1	6.3	6.1	6.3	6.1	6.4	6.1	6.4	6.1	6.2	6.1	6.1	6.1	6.1
2010	6.9	6.7	6.9	6.7	6.9	6.8	6.9	6.9	6.9	6.9	6.9	6.9	6.9	6.9
2011	7.4	7.7	7.4	7.6	7.4	7.7	7.4	7.8	7.4	7.7	7.4	7.7	7.5	7.6
2012	8.0	8.0	8.0	8.0	8.0	8.1	8.0	8.1	8.0	8.4	8.0	8.4	8.0	8.5
2013	8.5	8.6	8.5	8.6	8.5	8.5	8.5	8.4	8.5	8.4	8.5	8.5	8.5	8.4
2014	8.8	8.8	8.8	8.7	8.8	8.7	8.8	8.8	8.8	8.7	8.8	8.8	8.8	8.8
2015	9.1	9.2	9.1	9.1	9.1	9.3	9.1	9.3	9.1	9.1	9.1	9.1	9.1	9.1
2016	9.5	9.7	9.5	9.7	9.5	10.0	9.5	10.0	9.5	10.3	9.5	10.2	9.5	10.3
2017	9.9	9.8	9.9	9.8	9.9	9.8	9.9	9.8	9.9	9.7	9.9	9.7	10.0	9.7
**Comorbidity Score**														
Score 0	72.1	63.1	71.0	70.9	71.0	70.2	71.0	69.9	71.0	70.0	71.0	70.0	70.9	70.1

© 2023 Kucera CW et al. *JAMA Network Open.*

Score ≥1	27.9	37.0	29.0	29.1	29.0	29.8	29.0	30.1	29.0	30.0	29.0	30.0	29.1	29.9
**Neighbor Income**														
≥$63,333	38.4	20.9	38.3	21.0	36.3	36.1	36.3	36.0	36.3	36.6	36.3	36.8	36.2	36.9
$50,354 - $63,332	24.6	16.2	24.6	16.2	23.6	23.5	23.6	23.4	23.6	22.8	23.6	22.7	23.6	22.5
$40,227 - $50,353	22.6	19.2	22.7	19.2	22.2	22.3	22.2	22.3	22.2	22.3	22.2	22.3	22.2	22.3
<$40,227	14.4	43.7	14.4	43.6	17.9	18.1	18.0	18.4	18.0	18.3	18.0	18.2	18.0	18.3
**Insurance Status**														
Private	13.4	16.0	13.3	16.2	13.3	17.0	13.6	14.3	13.6	14.1	13.6	14.2	13.6	14.3
Medicare	85.3	78.1	85.3	77.8	85.3	77.0	84.4	83.6	84.5	83.8	84.5	83.7	84.5	83.6
Medicaid	1.0	4.6	1.0	4.5	1.0	4.3	1.4	1.6	1.4	1.5	1.4	1.5	1.4	1.5
Uninsured	0.4	1.4	0.4	1.5	0.4	1.6	0.5	0.6	0.6	0.6	0.5	0.6	0.6	0.6
**Histology / Grade (G)**														
Endometrioid G1	28.3	12.9	28.3	12.9	28.2	13.1	28.2	13.2	26.3	26.8	26.3	26.9	26.3	26.9
Endometrioid G2	22.1	13.2	22.1	13.3	22.1	13.4	22.1	13.4	20.9	21.6	20.9	21.8	20.9	21.7
Endometrioid G3	11.0	13.4	11.0	13.5	11.0	13.1	11.0	13.2	11.3	11.1	11.3	11.1	11.3	11.3
Endometrioid Gx	11.6	7.6	11.6	7.5	11.6	7.4	11.6	7.4	11.1	10.6	11.1	10.4	11.1	10.2
Mixed	6.2	6.5	6.2	6.4	6.2	6.4	6.2	6.4	6.2	6.2	6.2	6.2	6.2	6.2
Serous	8.7	20.4	8.7	20.6	8.7	21.0	8.7	20.9	10.2	10.0	10.2	10.0	10.2	10.0
Clear Cell	1.9	3.8	1.9	3.7	1.9	3.4	1.9	3.3	2.1	1.9	2.1	1.9	2.1	1.9
Carcinosarcoma	5.2	13.8	5.2	13.8	5.2	13.7	5.2	13.7	6.3	6.2	6.3	6.2	6.3	6.2
Other Cell Types	5.2	8.3	5.2	8.4	5.2	8.6	5.2	8.5	5.6	5.7	5.6	5.7	5.6	5.8
**Stage**														
I	70.7	52.3	70.7	52.2	70.6	53.3	70.5	53.5	69.2	64.5	68.4	69.0	68.4	69.1
II	6.7	8.9	6.7	8.9	6.7	8.6	6.7	8.5	6.8	7.7	6.9	6.8	6.9	6.8
III	13.6	19.4	13.6	19.7	13.7	19.3	13.7	19.2	14.2	14.5	14.3	13.7	14.3	13.6
IV	9.0	19.2	9.0	19.2	9.1	18.9	9.1	18.8	9.8	13.3	10.4	10.5	10.4	10.4
**Treatment**														
Surgical Treatment	92.5	83.5	92.5	83.6	92.4	83.6	92.4	84.4	92.2	85.4	92.0	86.8	91.3	91.0
No Surgery	7.5	16.5	7.5	16.4	7.6	16.4	7.6	15.6	7.8	14.6	8.0	13.2	8.7	9.0
Radiation Therapy	32.0	33.5	32.0	33.5	32.0	33.4	32.0	33.2	32.3	29.9	32.3	29.9	32.2	30.1
No Radiation	68.0	66.5	68.0	66.5	68.0	66.6	68.1	66.8	67.7	70.1	67.7	70.1	67.8	69.9
Chemotherapy	20.7	34.0	20.7	34.2	20.7	34.1	20.7	34.0	22.2	22.8	22.5	21.6	22.4	22.1
No Chemotherapy	79.3	66.1	79.3	65.8	79.3	65.9	79.4	66.0	77.8	77.2	77.6	78.4	77.7	77.9

[1] A propensity score balancing procedure was applied in seven steps to the original study population diagnosed at ≥65 years composed of 106,207 NHW and 14,815 NHB patients. **Step 1** balanced demographics, including age at diagnosis, census division, and year of diagnosis. **Step 2** extended balancing of demographic factors to include comorbidity score. **Steps 3** extended balancing of demographic and comorbidity factors to include neighborhood income.

© 2023 Kucera CW et al. *JAMA Network Open.*

Step 4 extended balancing of demographics, comorbidity score and neighborhood income to include insurance status. **Step 5** extended balancing of demographics, comorbidity score, neighborhood income, and insurance status to include histology. **Step 6** extended balancing of demographics, comorbidity score, neighborhood income, insurance status and histology to include stage. **Step 7** extended balancing of demographics, comorbidity score, neighborhood income, insurance status, histology, and stage to include treatment with surgery (no versus yes), radiation (no versus yes), and chemotherapy (no versus yes).

© 2023 Kucera CW et al. *JAMA Network Open.*

eTable 3. Sensitivity Analysis of Factors Associated With Risk of Death, Excess Relative Risk of Death, and Both the Percent Individual Contribution (IC) and the Cumulative Contribution to the Survival Disparities Between Non-Hispanic Black (NHB) and Non-Hispanic White (NHW) Patients With Uterine Cancer Diagnosed at All Ages, the Subset Diagnosed at <65 Years Old or the Subset Diagnosed at ≥65 Years Old by Applying a Sequential Propensity Score Balancing Procedure

**All Patients (Age 40-90)**		**HR (95% CI)[6]**	**ERR[7]**	**IC to ERR[8]**	**CC to ERR[8]**
Sequentially Balanced Variables[1]	Step 1. Demographic (baseline)[2]	2.029 (1.983-2.076)	1.029	-	-
	Step 2. Adding Histology[3]	1.423 (1.386-1.461)	0.423	58.9	58.9
	Step 3. Adding Stage[3]	1.306 (1.272-1.342)	0.306	11.4	70.3
	Step 4. Adding Comorbidity Score[4]	1.284 (1.250-1.319)	0.284	2.1	72.4
	Step 5. Adding Neighborhood Income[4]	1.238 (1.199-1.277)	0.238	4.5	76.9
	Step 6. Adding Insurance Status[4]	1.218 (1.180-1.257)	0.218	1.9	78.8
	Step 7. Adding Treatment[5]	1.182 (1.145-1.220)	0.182	3.5	82.3
	Unexplained	-	-	17.7	100
**Age <65 Years**		**HR (95% CI)[6]**	**ERR[7]**	**IC to ERR[8]**	**CC to ERR[8]**
Sequentially Balanced Variables[1]	Step 1. Demographic (baseline)[2]	2.429 (2.344-2.516)	1.429	-	-
	Step 2. Adding Histology[3]	1.567 (1.505-1.631)	0.567	60.3	60.3
	Step 3. Adding Stage[3]	1.428 (1.372-1.487)	0.428	9.7	70.0
	Step 4. Adding Comorbidity Score[4]	1.407 (1.351-1.466)	0.407	1.5	71.5
	Step 5. Adding Neighborhood Income[4]	1.363 (1.301-1.429)	0.363	3.1	74.6
	Step 6. Adding Insurance Status[4]	1.308 (1.247-1.372)	0.308	3.8	78.4
	Step 7. Adding Treatment[5]	1.291 (1.231-1.353)	0.291	1.2	79.6
	Unexplained	-	-	20.4	100
**Age ≥65 Years**		**HR (95% CI)[6]**	**ERR[7]**	**IC to ERR[8]**	**CC to ERR[8]**
Sequentially Balanced Variables[1]	Step 1. Demographic (baseline)[2]	**1.868 (1.812-1.926)**	0.868	-	-
	Step 2. Adding Histology[3]	**1.364 (1.315-1.415)**	0.364	58.1	58.1
	Step 3. Adding Stage[3]	**1.255 (1.210-1.303)**	0.255	12.6	70.7
	Step 4. Adding Comorbidity Score[4]	**1.233 (1.187-1.280)**	0.233	2.5	73.2
	Step 5. Adding Neighborhood Income[4]	**1.199 (1.147-1.254)**	0.199	3.9	77.1
	Step 6. Adding Insurance Status[4]	**1.197 (1.144-1.253)**	0.197	0.2	77.3
	Step 7. Adding Treatment[5]	**1.137 (1.087-1.190)**	0.137	6.9	84.2
	Unexplained	-	-	15.8	100

1 Propensity score analysis was applied to sequentially balance the following compliment of clinical covariates in NHB and NHW patients in a seven-step process moving histology and stage at diagnosis up after the demographic factors at birth, and positioning comorbidity score, neighborhood income and insurance down in front of first-line treatment.

2 Step 1 balanced demographics, including age at diagnosis, census division, and year of diagnosis at birth in the baseline model.

3 Steps 2 and 3 balanced factors at diagnosis. Step 2 extended balancing of demographic factors to include histology. Steps 3 extended balancing of demographic and histology to include stage.

4 Steps 4 through 6 balanced factors prior to diagnosis. Step 4 extended balancing of demographics, histology, and stage to include comorbidity score. Step 5 extended balancing of demographics, histology, stage, and comorbidity score to include neighborhood income. Step 6 extended balancing of demographics, histology, stage, comorbidity score and neighborhood income to include insurance status.

5 Step 7 extended balancing of demographics, histology, stage, comorbidity score, neighborhood income, and insurance status to include factors regarding first-line treatment utilization with surgery (no versus yes), radiation (no versus yes), and chemotherapy (no versus yes).

© 2023 Kucera CW et al. *JAMA Network Open.*

[6] Weighted Cox modeling was performed to estimate the hazard ratio (HR) and the 95% confidence interval (CI) in the baseline model at birth with adjustments for age, census division, and year of diagnosis, and then after each sequential balancing step.

[7] The total excess relative risk (ERR) of death in NHBs relative to NHWs was calculated by subtracting one from the baseline adjusted HR.

[8] The contribution of individual factors (IC) and the cumulative contribution (CC) to the survival difference between NHB and NHW patients were calculated based on the reduction in HR after sequential adjustment for the additional covariates. The proportion of total ERR explained by each factor derived as: $[(HR_b - HR_a)/(1 - HR_{baseline})] \times 100\%$, where HR_b and HR_a indicating the HR estimated before and after balance of the corresponding factor, and $HR_{baseline}$ indicating the HR from baseline model.

© 2023 Kucera CW et al. *JAMA Network Open.*

eFigure 1. Schema for Selection of Study Population

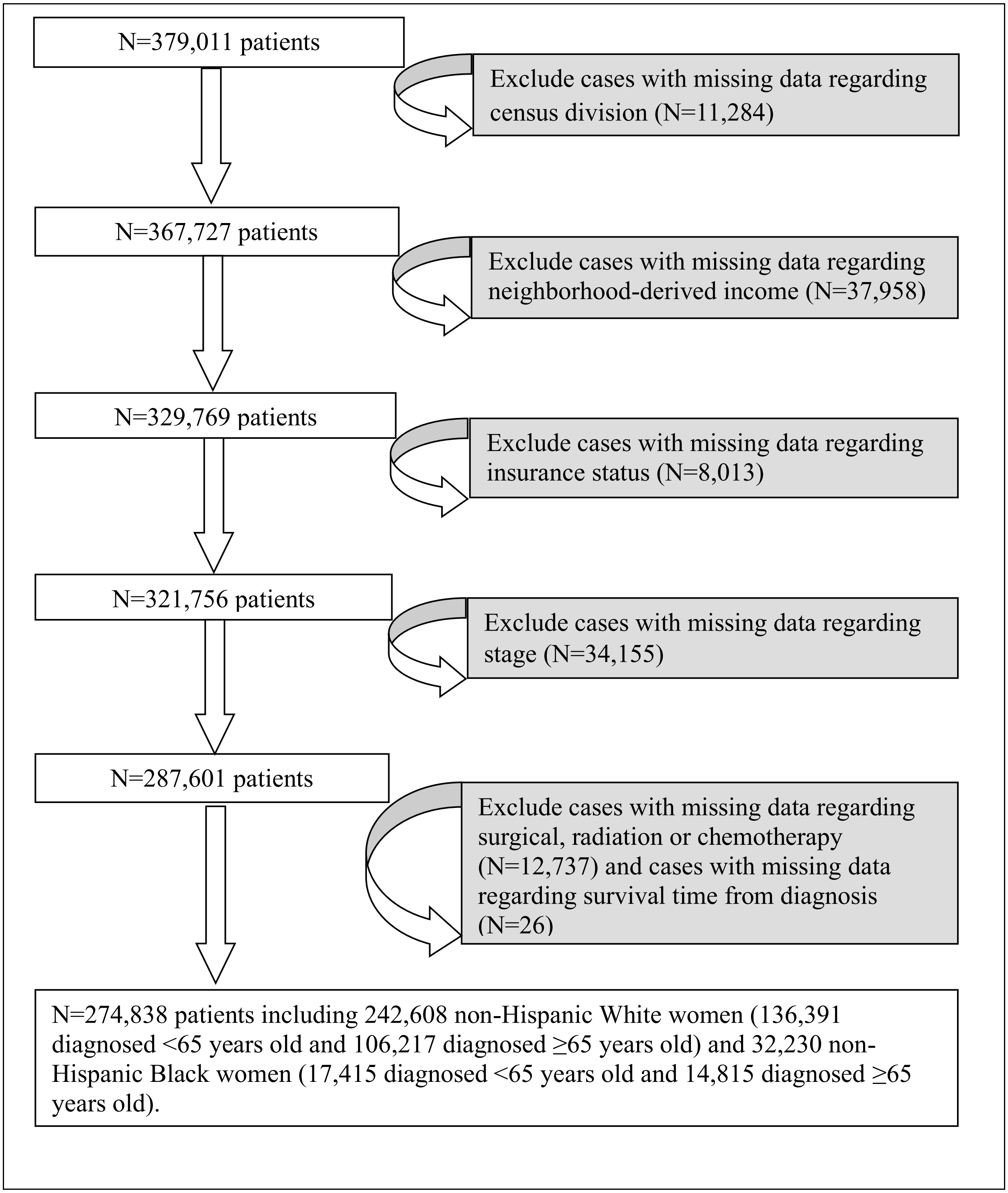

© 2023 Kucera CW et al. *JAMA Network Open.*

eFigure 2. The Distribution of Clinical Characteristics Associated With Poor Prognosis in Non-Hispanic White (NHW, Blue) vs Non-Hispanic Black (NHB, Red) Patients With Uterine Cancer

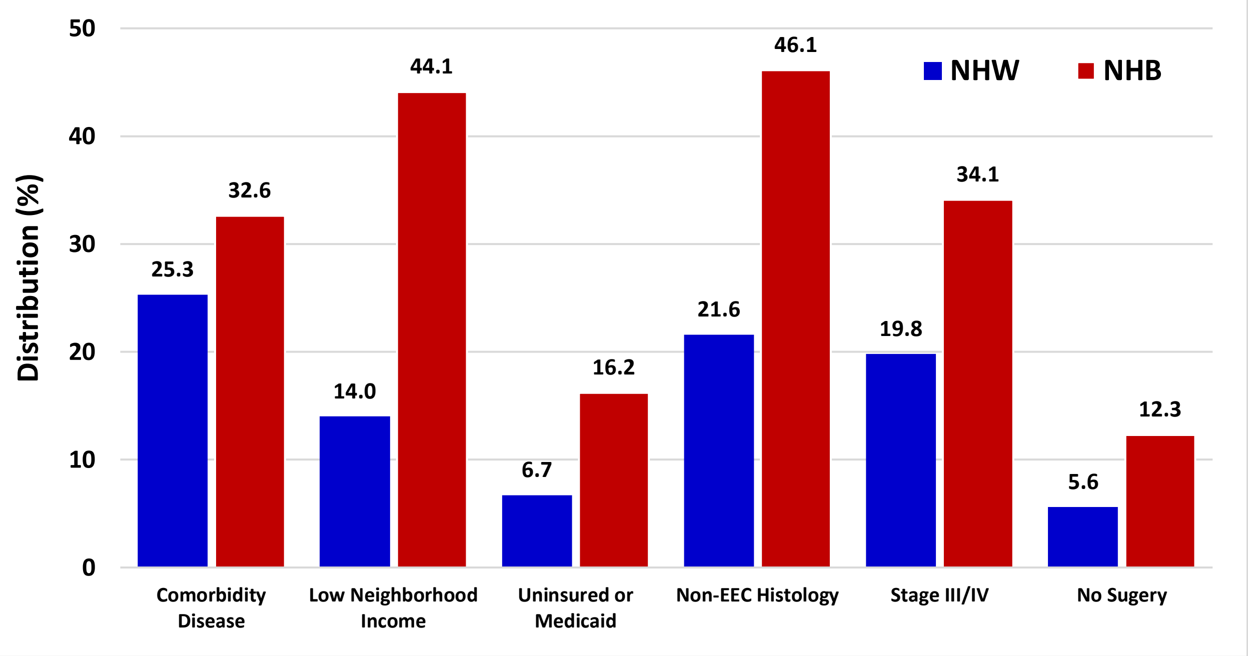

© 2023 Kucera CW et al. *JAMA Network Open.*

eFigure 3. The distribution of Insurance Status in Non-Hispanic White (NHW, Blue) vs Non-Hispanic Black (NHB, Red) Patients With Uterine Cancer Stratified by Age at Diagnosis

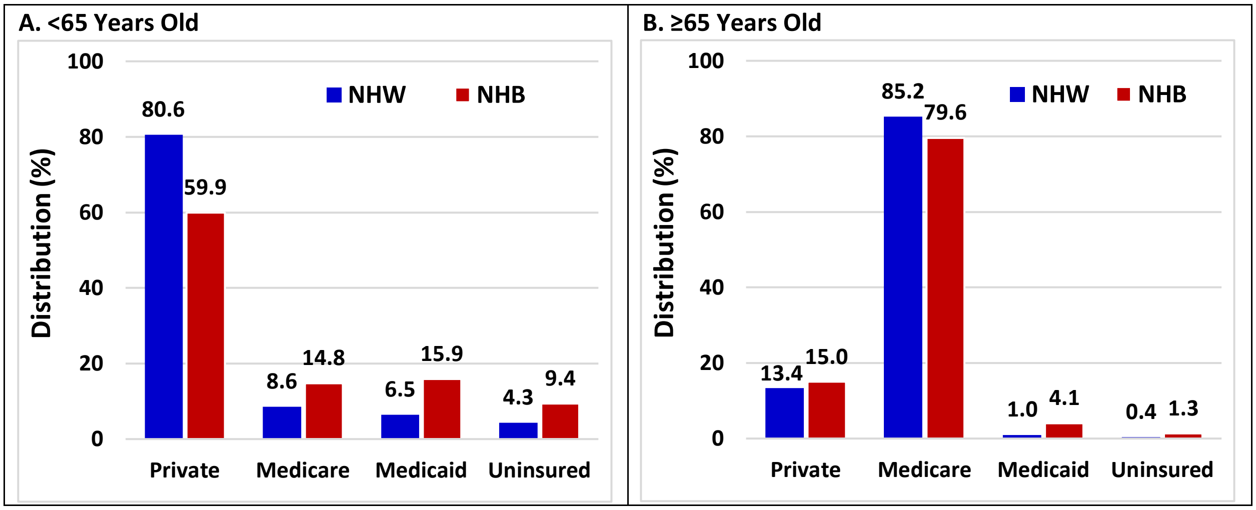

© 2023 Kucera CW et al. *JAMA Network Open.*
