# Supplementary material for: Factors Associated With Survival Disparities Between Non-Hispanic Black and White Patients With Uterine Cancer
Source: JAMA Netw Open. 2023 Apr 17;6(4):e238437. doi: 10.1001/jamanetworkopen.2023.8437 (PMC10111180; doi:10.1001/jamanetworkopen.2023.8437)
Supplement: Supplement 2. — Data Sharing Statement [file jamanetwopen-e238437-s002.pdf]

Data Sharing Statement

Kucera. Factors Associated With Survival Disparities Between Non-Hispanic Black and White Patients With Uterine Cancer. *JAMA Netw Open*. Published April 17, 2023. doi:10.1001/jamanetworkopen.2023.8437

Data
Data available: No

Additional Information
Explanation for why data not available: The data for this study was acquired by application to the restricted access National Cancer Database and the data sharing agreement precludes data sharing. These details are declared in the data sharing statement section of this manuscript.
